# Different AMPA receptor subtypes mediate the distinct kinetic components of a biphasic EPSC in hippocampal interneurons

**DOI:** 10.3389/fnsyn.2015.00007

**Published:** 2015-05-18

**Authors:** Todd L. Stincic, Matthew E. Frerking

**Affiliations:** ^1^Casey Eye Institute, Oregon Health and Science UniversityPortland, OR, USA; ^2^Department of Behavioral Neuroscience, Oregon Health and Science UniversityPortland, OR, USA

**Keywords:** GluA2, hippocampus, AMPA receptor, stratum radiatum, stratum lacunosum moleculare

## Abstract

CA1 hippocampal interneurons at the border between stratum radiatum (SR) and stratum lacunosum-moleculare (SLM) have AMPA receptor (AMPAR)-mediated excitatory postsynaptic currents (EPSCs) that consist of two distinct phases: a typical fast component (FC), and a highly unusual slow component (SC) that persists for hundreds of milliseconds. To determine whether these kinetically distinct components of the EPSC are mediated by distinct AMPAR subpopulations, we examined the relative contributions of GluA2-containing and—lacking AMPARs to the SC. GluA2-containing AMPARs mediated the majority of the FC whereas GluA2-lacking AMPARs preferentially generated the SC. When glutamate uptake through the glial glutamate transporter excitatory amino acid transporter (EAAT1) was inhibited, spill over-mediated AMPAR activation recruited an even slower third kinetic component that persisted for several seconds; however, this spillover-mediated current was mediated predominantly by GluA2-containing AMPARs and therefore was clearly distinct from the SC when uptake is intact. Thus, different AMPAR subpopulations that vary in GluA2 content mediate the distinct components of the AMPAR EPSC. The SC is developmentally downregulated in mice, declining after the second postnatal week. This downregulation affects both GluA2-containing and GluA2-lacking AMPARs mediating the SC, and is not accompanied by developmental changes in the GluA2 content of AMPARs underlying the FC. Thus, the downregulation of the SC appears to be independent of synaptic GluA2 expression, suggesting the involvement of another AMPAR subunit or an auxiliary protein. Our results therefore identify GluA2-dependent and GluA2-independent determinants of the SC: GluA2-lacking AMPARs preferentially contribute to the SC, while the developmental downregulation of the SC is independent of GluA2 content.

## Introduction

Local inhibitory interneurons control activity in the hippocampus by tightly regulating the excitability and synchrony of hippocampal circuits (Freund and Buzsáki, 1996; McBain and Fisahn, 2001; Baraban and Tallent, 2004). These functions require that interneuronal activation occurs with high temporal precision. Consequently much of the current research has emphasized the rapid kinetics associated with the AMPA/kainate receptor (AMPAR/KAR)-mediated excitatory postsynaptic currents (EPSCs) on these cells (McBain et al., 1999). Surprisingly, a biphasic AMPAR/KAR EPSC lasting for several hundred milliseconds has been observed in interneurons along the border between stratum radiatum (SR) and stratum lacunosum-moleculare (SLM) in area CA1 (Frerking et al., 1998). Careful examination of the EPSC on these cells revealed the typical fast component (FC) was mediated entirely by AMPARs, whereas the unusual slow component (SC) was generated by AMPARs and GluK1-containing KARs in roughly equal proportions (Wondolowski and Frerking, 2009). It must be noted that this SC of the EPSC is selectively expressed by the SR/SLM subpopulation and not found in interneurons of stratum oriens (Goldin et al., 2007; Wondolowski and Frerking, 2009). Using a combination of physiological recordings and modeling, the SC was shown to account for a significant portion of the charge transfer and sufficient to alter the function of this circuit from that of temporal coding to rate coding (Frerking and Ohliger-Frerking, 2002).

Slow KAR EPSCs have been observed and are thought to result from incorporation of a high affinity subunit, GluK4 or GluK5 (Lerma, 2003) into the heteromeric KAR complex, but no analogous AMPAR subunit exists and the SC of the AMPAR EPSC is unexpected given AMPAR desensitization and deactivation rates rest in the millisecond range (Jonas and Spruston, 1994). One possible mechanism to explain the slow AMPAR kinetics is through a glutamate transient generated by spillover onto extrasynaptic receptors, and in support of this idea the slow AMPAR EPSC is selectively enhanced with glutamate spillover induced by the excitatory amino acid transporter (EAAT) inhibitor DL-threo-β-Benzyloxyaspartic acid (TBOA; Wondolowski and Frerking, 2009). However, the fast and SCs of the AMPAR EPSCs generated by stimulus trains summate linearly when uptake is intact, displaying no sign of frequency or stimulus number dependency that would suggest accumulating glutamate spillover (Frerking et al., 1998; Wondolowski and Frerking, 2009). Therefore, the mechanisms underlying the SC of the AMPAR EPSC may differ depending on the integrity of glutamate uptake. These findings suggest three AMPAR subpopulations mediate different portions of the EPSC: the FC, the SC when uptake is intact, and the SC when uptake is compromised. However, the mechanisms by which these subpopulations acquire their distinctive biophysical characteristics remain unknown.

While AMPARs are found throughout the brain, subunit expression varies by region (Martin et al., 1993). Importantly, the subunit composition of AMPARs affects kinetics and interactions with scaffolding and auxiliary proteins (Swanson et al., 1997; Shepherd and Huganir, 2007). Thus, distinct AMPARs complexes could potentially delineate the subgroups responsible for each component of the EPSC. Functional AMPARs are tetramers formed from GluA1–4 subunits in either homo- or hetero-meric arrangements, with the inclusion of the GluA2 subunit causing AMPARs to become impermeable to Ca^2+^ and resistant to polyamine toxins that block AMPARs lacking GluA2 (Greger et al., 2003; Mayer, 2005). Interneurons express a heterogeneous mix of GluA2-containing and GluA2-lacking AMPARs (Geiger et al., 1995; Petralia et al., 1997; Catania et al., 1998; He et al., 1998; Tóth and McBain, 1998; Moga et al., 2003; Szabo et al., 2012) which might contribute differently to the distinct kinetic components of the EPSC. In the present study, we test this hypothesis by comparing the kinetics and characteristics of pharmacologically-isolated GluA2-containing and GluA2-lacking AMPAR EPSCs in SR/SLM interneurons.

## Materials and Methods

Experimental procedures were in accordance with the National Institutes of Health guidelines for animal use and the protocols were approved by the Institutional Animal Care and Use Committee at OHSU. C57B/6 mice of either sex from Charles River (Wilmington, MA), aged 1–3 weeks, were anesthetized with halothane/isofluorane and sacrificed by rapid decapitation. Brains were dissected out and submerged in ice cold cutting solution consisting of (in mM): 110 Choline Cl, 7 MgCl_2_, 2.5 KCl, 1.25 NaH_2_PO_4_, 0.5 CaCl_2_, 25 NaHCO_3_, 1.3 Na ascorbate, 10 glucose, and bubbled with 95% O_2_/5% CO_2_. The most rostral and caudal portions of the brain were removed with a razor blade. Remaining tissue was affixed to the stage with cyanoacrylate glue and agarose. Rostral coronal sections, 300–400 μm, were taken at the level of the hippocampus with a Vibratome. Slices were kept at 37°C in cutting solution for 20 min, next cooled at room temperature for 20 min, and then transferred at least 30 min prior to recordings into a solution containing (in mM): 119 NaCl, 26.2 NaHCO_3_, 1 NaH_2_PO_4_, 2.5 KCl, 11 glucose, 4 MgSO_4_, 4 CaCl_2_ bubbled with 95% O_2_/5% CO_2_. Patch electrodes (2–5 MΩ) were filled with a solution adjusted to pH 7.2, 270–290 mOsm containing (in mM): 110 CsMeS, 5 QX314-Cl, 5 Cs-BAPTA, 10 HEPES, 4 Mg-ATP, 0.4 Tris-GTP, 10 Tris-Phosphocreatine, and 0.1 spermine. In all experiments antagonists were used to block NMDARs (100 μM D-AP5), GABA_A_Rs (100 μM picrotoxin), and GluK1-containing KARs (10 μM UBP 302), which we have found in previous studies to be sufficient to abolish the NMDAR EPSC, GABA_A_R IPSC, and KAR EPSC respectively (Frerking et al., 1998; Wondolowski and Frerking, 2009). Philanthotoxin-343 (PhTx; 1 μM, Sigma-Aldrich) was bath applied to block GluA2-lacking AMPARs, and NBQX (100 μM) was applied at the end of experiments to confirm that the recorded EPSC was glutamatergic. This high dose of NBQX was chosen as to assure all remaining glutamatergic signaling was abolished. Before additional recordings were performed the chamber was thoroughly flushed and a new slice was selected.

Stimulation and recording techniques were similar to those previously described (Frerking et al., 1998; Wondolowski and Frerking, 2009). Schaffer collateral/commissural fibers were stimulated with a bipolar stimulating electrode placed in SR. Whole-cell patch clamp recordings were made by visual identification of interneurons clustered around the SR/SLM border using IR-DIC microscopy and voltage clamped at −70 mV. Cells with a characteristic pyramidal shape or large dendritic branches sent out toward stratum lacunosum were avoided, as these might be a subpopulation of pyramidal cells with soma in radiatum (Klausberger and Somogyi, 2008).

Electrophysiological recordings were obtained with an Axoclamp 200B amplifier and IgorPro software, filtered at 2 kHz, and digitized at 5 kHz. Series resistances (typically between 10 and 25 MΩ and input resistances (typically between 200 and 500 MΩ were monitored online to ensure stability of recordings. Recordings were excluded from analysis if these parameters changed by >25% over the course of the experiment. Cells were also excluded if the observed result could be explained by an associated change in either parameter, even if the magnitude of the change was <25%.

After sufficient time passed post-patch to allow for equilibration of internal solution, 5 pulses were delivered at 30 Hz every 14 s throughout the course of the experiment. The amplitude of the first peak in the pulse train was continuously monitored during the drug applications to assess when a stable drug effect was achieved. Averaged EPSCs were constructed before and after drug applications using 20–25 sweeps. The average sweep in NBQX was subtracted from both baseline and PhTx averages to ensure that the recorded currents were due to glutamatergic transmission. Pulse trains were used both to monitor changes in short-term plasticity as well as improve discrimination of the tail current.

Data analysis was performed using IgorPro 5 and SigmaPlot 11 software. Data were compared using the Student’s *t* test, Rank Sum test, or ANOVA as appropriate based on the comparisons being made and whether or not the data were distributed normally. Significance was assessed at *p* < 0.05. All data are presented as mean ± SEM, regardless of normality of distribution, to aid in direct visual comparison. Fits were calculated through the dynamic fitting routine of Sigmaplot.

## Results

### GluA2-Containing AMPARs Preferentially Contribute to the Slow Component

Previous work has established that hippocampal interneurons express AMPARs both containing and lacking the GluA2 subunit (Geiger et al., 1995; Kullmann and Lamsa, 2007) and that these receptor subtypes can be separately trafficked to distinct locations (Tóth and McBain, 1998). To determine whether GluA2-containing and GluA2-lacking AMPARs differentially contribute to the fast and SCs of the EPSC, we examined the effects of the GluA2-lacking AMPAR inhibitor PhTx on the AMPAR ESPC.

**Figure 1:**
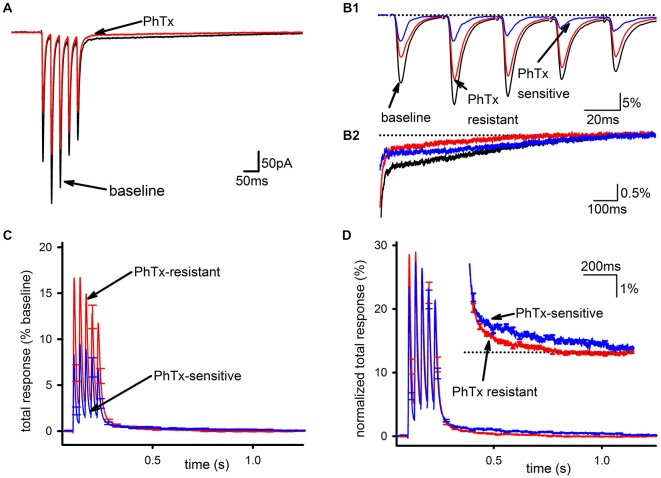
**GluA2-lacking AMPARs are the main contributor to the slow EPSC in hippocampal interneurons. (A)** Representative traces of an average EPSC before and after bath application of PhTx. **(B1)** Isolation of PhTx-resistant (red) and—sensitive (blue) currents overlaid with the current prior to addition of PhTx and normalized to the summed release during the pulse train in baseline conditions. **(B2)** An expanded view of the tail current is shown. **(C)** PhTx resistant and sensitive currents were normalized to the total response in the absence of PhTx, and averaged together across multiple cells. Normalization by the total response, defined as defined as the peak response to each stimulus during the train after subtraction of the current immediately preceding that stimulus, summed together over all five stimuli, allows a direct comparison of the slow components in each condition, as this component is the summed response to all five stimuli during the train. **(D)** PhTx-resistant and—sensitive currents are shown re-normalized to the total response for each respective condition individually, which allows a direct comparison of the kinetics of the slow components in each condition. Inset is an expanded view of tail demonstrating the sensitive current has a slower decay phase.

PhTx had a clear but partial inhibitory effect on the EPSC, as shown in Figure 1A (baseline EPSC; EPSC in PhTx) and assessed by the reduction in total synaptic charge transfer (*n* = 24, 39 ± 4% inhibition, *p* < 0.001). This inhibition was not accompanied by any effect on short-term plasticity of the EPSC as assessed by the amplitude ratio for the 5th and 1st EPSCs in the train (*n* = 24, baseline: 0.841 ± 0.19; PhTx: 0.79 ± 0.18, *p* > 0.05), confirming that the effect of PhTx was postsynaptic. The PhTx-resistant portion of the EPSC, representing GluA2-containing AMPARs, was measured directly as the EPSC following PhTx application; the PhTx-sensitive portion, representing GluA2-lacking AMPARs, was calculated (blue traces) by subtracting the EPSC in PhTx from that in baseline conditions (Figure 1B). Inspection of the slow EPSC following the last pulse in the stimulus train (Figure 1B) shows that the PhTx-sensitive current accounts for nearly all of the tail current after a few hundred milliseconds.

To compare the PhTx-sensitive and PhTx-resistant EPSCs across recordings, EPSCs from individual cells were examined after normalizing to total baseline response, calculated as the sum of all five EPSC peak amplitudes during the stimulus train (Figure 1C). This was necessary to account for experimental conditions where changes in the SC could profoundly influence the apparent amplitude of the FC of EPSCs late in the train due to temporal summation. The majority of AMPAR current during the FC was PhTx-resistant, while the tail was mostly composed of the PhTx-sensitive current. Normalizing the PhTx-resistant and PhTx-sensitive EPSCs each to their own respective amounts of total response allowed us to directly compare kinetics of the PhTx-resistant and—sensitive EPSCs; Figure 1D shows these two currents do not scale proportionally, with the PhTx-sensitive EPSC being much slower than the PhTx-resistant EPSC. However, it is also clear that both the PhTx-sensitive and—resistant EPSCs have a biphasic decay, necessitating a more quantitative analysis.

**Figure 2:**
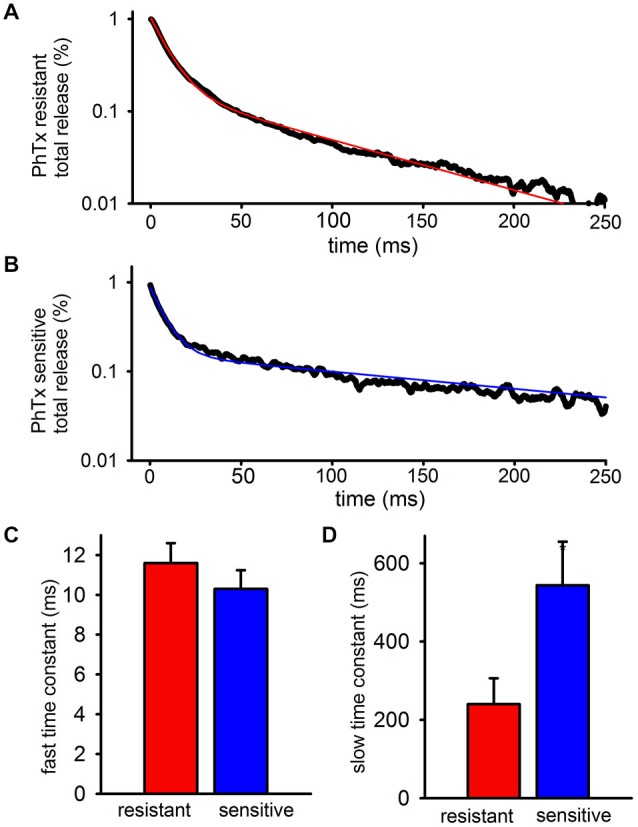
**GluA2-lacking AMPARs contributing to the slow EPSC have slower decay kinetics than GluA2-containing AMPARs contributing to the slow EPSC**. The PhTx- resistant and—sensitive currents after the final peak were isolated, fitted, and averaged. **(A)** Bi-exponential fit (red) plotted over representative data for a PhTx resistant current. **(B)** Bi-exponential fit (blue) plotted over representative data for a PhTx sensitive current. **(C)** Graph showing the average fast time constant is no different for PhTx resistant and sensitive currents. **(D)** Graph showing that the average slow time constant for the PhTx sensitive current is significantly faster than the resistant current.

To quantify the kinetics of each EPSC more precisely, the decay phases of the PhTx-resistant (Figure 2A) and—sensitive (Figure 2B) EPSCs after the fifth stimulus pulse of the train were isolated and fit to the sum of two exponentials. The decay of the FC of the EPSC was not resolvably different between PhTx-resistant EPSCs and PhTx-sensitive EPSCs (Figure 2C: *n* = 24, PhTx-resistant, 11.6 ± 1 ms; PhTx-sensitive 10.3 ± 0.9 ms; *p* > 0.05). Similarly, the relative contribution of the fast exponential to the EPSC decay was not significantly different for the PhTx-sensitive and PhTx–resistant EPSCs (*n* = 24, PhTx-resistant: 86 ± 2.4%; PhTx-sensitive: 87 ± 1.9%, *p* > 0.05; not shown). However, the average time constant for the SC of the PhTx-sensitive EPSC was much slower than that of the PhTx-resistant EPSC (Figure 2D: *n* = 24, PhTx-resistant: 240.3 ± 66 ms; PhTx-sensitive: 544 ± 111 ms; *p* < 0.05). Thus, both GluA2-lacking and GluA2-containing AMPARs both contribute to the fast EPSC and slow EPSC, and the fast EPSC mediated by each AMPAR subpopulation has similar kinetics; however, the slow EPSC mediated by GluA2-lacking AMPARs is much slower than the slow EPSC mediated by GluA2-containing AMPARs. As a result, after a few hundred milliseconds the SC of the EPSC is mediated almost entirely by GluA2-lacking AMPARs.

### EAAT1 Limits Spillover of Stimulated Glutamate Release

We have previously shown that an impairment of glutamate uptake can profoundly increase the size of the slow AMPAR-mediated EPSC, although it remains unclear whether this is a stronger activation of the same receptors that produce the slow EPSC when uptake is intact, or recruitment of a distinct population of AMPARs (Wondolowski and Frerking, 2009). To address this point, we decided to more specifically examine the mechanism and effects of blocking uptake on the components of the interneuronal EPSC.

EAATs, localized both to neurons and glia, are responsible for glutamate uptake. EAATs 1–3 are expressed in the central nervous system where EAAT3 is neuronal and EAATs 1 and 2 are glial (O’Shea, 2002; Beart and O’Shea, 2007). At the high concentration (100 μM) used in our prior study, TBOA blocks all three isoforms. In order to determine which subtypes are active at this particular synapse, we applied the EAAT2-selective blocker WAY213613 (0.5 μM), TBOA at a low dose (10 μM) to preferentially block EAAT3 in addition to EAAT2, or TBOA at a high dose (100 μM) to additionally block EAAT1. In most experiments, all three of these drugs were applied sequentially.

**Figure 3:**
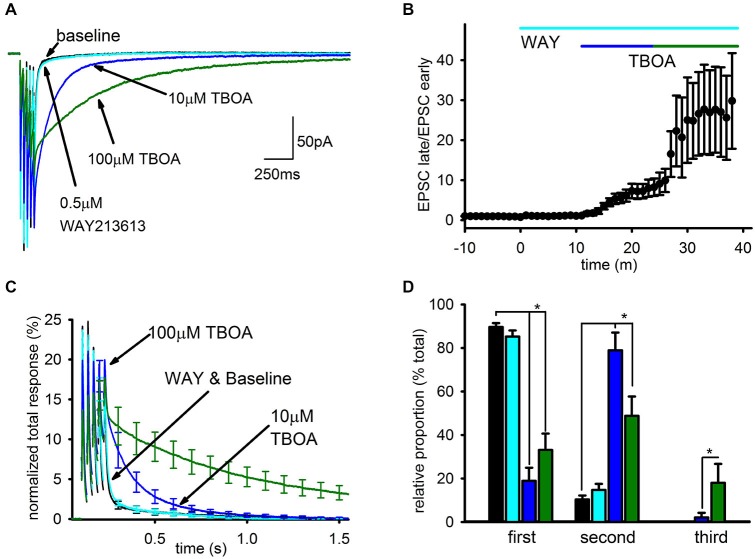
**Blockade of EAAT1 and EAAT3 recruits a spillover- mediated slow AMPAR tail. (A)** Representative average traces are shown for baseline and subsequent application of each drug. **(B)** The change in average ratio of the late EPSC relative to the charge transfer for the early EPSC is shown normalized to baseline. **(C)** Average group EPSCs normalized to baseline total response are shown after addition of each drug. 0.5 μM WAY213613 had a minimal effect while 10 mM TBOA had an appreciable observed enhancement of the tail, suggesting EAAT1 is the major glutamate transporter at this synapse. **(D)** Exponential fitting found that both 10 and 100 μM TBOA increased the relative proportion of the slow component. However, only the highest dose reliably recruited a third, very slow current.

Representative traces can be seen in Figure 3A depicting the EPSC after addition of each EAAT inhibitor, and the time-course of the enhancement of the slow EPSC caused by the different inhibitors is shown in Figure 3B. WAY213613 had no significant effect on the charge transfer of the SC compared to baseline (*n* = 5, baseline: 94.8 ± 8%; WAY213613: 93.7 ± 12%; *p* > 0.5), while 10 μM TBOA approximately doubled the total charge transfer (*n* = 6, 215 ± 35.8%, *p* < 0.05), predominantly due to an increase in the relative size, but not kinetics, of the slow EPSC. As a result, the EPSC in 10 μM TBOA EPSC was almost entirely complete after ~1 s. The subsequent addition of 100 μM TBOA profoundly enhanced the slow EPSC as seen in our prior study, leading to a large increase in the total charge transfer (*n* = 7, 100 μM TBOA: 831 ± 173%) and a substantial slowing of the EPSC decay so that the EPSC persisted for several seconds after the final train pulse as seen in Figure 3C.

To quantitate these observations, the decay of the EPSC after the final pulse in the train was fit by 3 exponentials: τ_1_ was constrained to a time constant of <50 ms to fit the FC, τ_2_ was constrained to a time constant of between 50 ms and 1 s to fit the SC seen in baseline conditions, and τ_3_ was constrained to a time constant >1 s to fit the SC recruited by TBOA. The relative contributions of each component to the EPSC were profoundly affected by uptake inhibition (Figure 3D). The SC of the EPSC associated with τ_2_ was significantly enhanced in both doses of TBOA relative to baseline and WAY213613 conditions (baseline: *n* = 7, 10 ± 2%; WAY213613: *n* = 5, 15 ± 3%; 10 μM TBOA: *n* = 6, 58 ± 12%; 100 μM TBOA: *n* = 7, 49 ± 9%, *p* < 0.01); additionally, the slowest component of the EPSC associated with τ_3_ was only observed in the presence of TBOA, and was only significantly larger than baseline conditions at 100 μM TBOA (baseline: *n* = 7, 0 ± 0%; WAY213613: *n* = 5, 0 ± 0%; 10 μM TBOA: *n* = 6, 1 ± 1%; 100 μM TBOA: *n* = 7, 18 ± 9% *p* < 0.05). The increases in the relative proportion of slow EPSCs in TBOA came at the expense of the relative proportion of the fast EPSC (baseline: *n* = 7, 90 ± 2%; WAY213613: *n* = 5, 85 ± 3%; 10 μM TBOA: *n* = 6, 41% ± 12%; 100 μM TBOA: *n* = 7, 33 ± 8% *p* > 0.05).

In contrast, the time constants for each exponential were largely unaffected by the different conditions. The only resolvable difference in the time constant of decay for any of the EPSC components in any of the conditions was a statistically significant increase in the duration of the second decay component in 100 μM TBOA (τ_1_ not shown; τ_2_ in baseline: *n* = 7, 286 ± 78 ms, WAY213613: *n* = 5, 244 ± 55 ms; 10 μM TBOA: *n* = 6, 296 ± 65 ms; 100 μM TBOA: *n* = 7, 606 ± 97 ms, *p* < 0.05; τ_3_ in baseline: N/A, WAY213613: N/A, 100 μM TBOA: 2373 ± 265 ms); however, even in this case we caution that this finding may be a result of difficulties in unambiguously separating the second and third exponents in this condition.

Thus, the overwhelming majority of glutamate uptake that limits the spread of glutamate at these synapses is mediated by EAAT1, inhibition of which leads to the recruitment of a very SC of the EPSC that is not substantively present when EAAT1 retains function. In contrast, EAAT3 has a minor but resolvable role that limits the size of the slow EPSC seen in baseline conditions, while EAAT2 has no apparent effect on the EPSC at these synapses.

### The Slow EPSC Recruited by TBOA is not Preferentially Mediated by GluA2-Lacking AMPARs

From the experiments in Figures 1, 2, we concluded that GluA2-lacking AMPARs contribute disproportionately to the slow EPSC when uptake is intact. Because 100 μM TBOA recruits an even slower EPSC than seen in baseline conditions, we wondered whether GluA2-lacking AMPARs would also disproportionately mediate this current. If this were the case, it might suggest that the SC under baseline conditions reflects a modest recruitment of extrasynaptic GluA2-lacking AMPARs, which can be profoundly enhanced by limiting glutamate uptake. Alternatively, if the EPSC recruited by TBOA is not predominantly mediated by GluA2-lacking AMPARs, this would argue that the subpopulation of AMPARs recruited by TBOA is distinct from the subpopulation of AMPARs that generate the slow AMPAR EPSC under baseline conditions. We therefore examined the effects of PhTx on EPSCs in the continuous presence of 100 μM TBOA.

**Figure 4:**
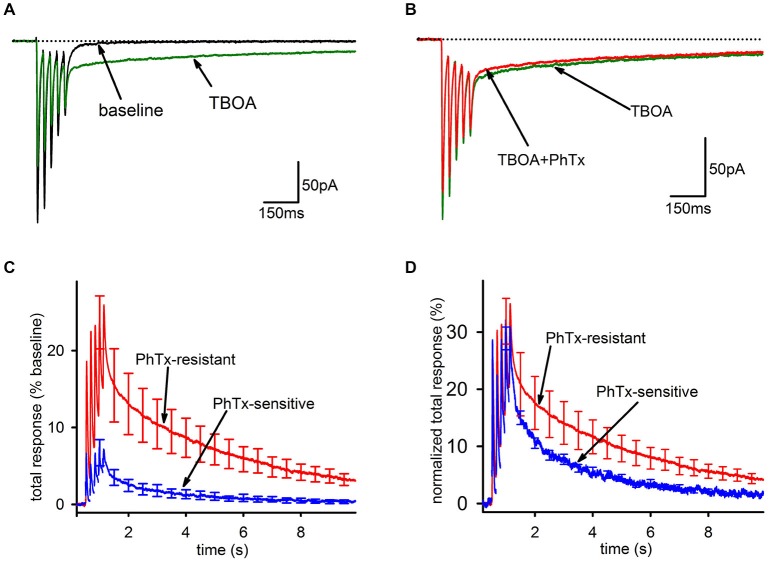
**Blockade of EAAT1 recruits extrasynaptic AMPARs that predominantly contain GluA2. (A)** Representative traces of before (black) and after addition of TBOA (green). TBOA results in a small reduction of the fast component and a major enhancement of the tail current. **(B)** Representative traces of the EPSC in TBOA, before (green) and after (red) PhTx. **(C)** Group average PhTx-resistant (red) and—sensitive (blue) currents are shown normalized to total response in TBOA. The PhTx-sensitive current is only a small portion of the total current and does not preferentially contribute to the tail. **(D)** Group average PhTx-resistant (red) and—sensitive (blue) currents are shown normalized to their own total response in order to compare kinetics. A bi-exponential fit found no significant differences for either the relative proportions of the current or the time constants of the two currents.

As expected, bath application of 100 μM TBOA caused a modest decrease in the FC, likely mediated by AMPAR desensitization from increased ambient glutamate. Further, there was a massive increase in the SC, leading to a greatly enhanced overall charge transfer (Figure 4A). However, in stark contrast to the result expected if glutamate spillover predominantly recruits GluA2-lacking AMPARs, PhTx had a smaller inhibitory effect on the total AMPAR-mediated charge transfer in the presence of TBOA than it did under baseline conditions (Figure 4B, baseline: *n* = 25, 44 ± 6%; TBOA: *n* = 8, 18 ± 6%; *p* < 0.05), reflecting a greater contribution of GluA2-containing AMPARs in the presence of TBOA than in its absence. As a percentage of the total integral in TBOA, the PhTx-resistant EPSC was predominant (Figure 4C, *n* = 8, PhTx-resistant, 85 ± 8%; PhTx-sensitive, 15 ± 7%). When the kinetics of the PhTx-resistant EPSC in TBOA were directly compared to those of the PhTx-sensitive EPSC in TBOA, the decay of the PhTx-sensitive EPSC was not clearly slower than that of the PhTx-resistant EPSC (Figure 4D); a tri-exponential fit failed to reveal any significant differences between the decay time constants of the fast (*n* = 8, 9.8 ± 1.5 ms; *n* = 6, 12.8 ± 2.4 ms; *p* = 0.622), slow (*n* = 8, 570 ± 106 ms; *n* = 6, 485 ± 195 ms, *p* = 0.691), or uptake-suppressed (*n* = 8, 2489 ± 395 ms; *n* = 4, 1497 ± 474 ms; *p* > 0.05) components of the PhTx-resistant and—sensitive currents of the TBOA-enhanced tail. There was also no difference between the relative proportions of the fast (*n* = 8, 0.5 ± 0.08; *n* = 6, 0.46 ± 0.08; *p* > 0.05), slow (*n* = 8, 0.29 ± 0.08; *n* = 6, 0.36 ± 0.09; *p* > 0.05), or uptake-suppressed (*n* = 8, 0.21 ± 0.05; *n* = 6, 0.19 ± 0.07; *p* > 0.05) EPSCs. We conclude that the slow EPSC recruited by TBOA is not composed predominantly of GluA2-lacking AMPARs, and in this respect it is clearly distinct from the slow EPSC when uptake is intact.

### The Slow AMPAR EPSC is Developmentally Downregulated

**Figure 5:**
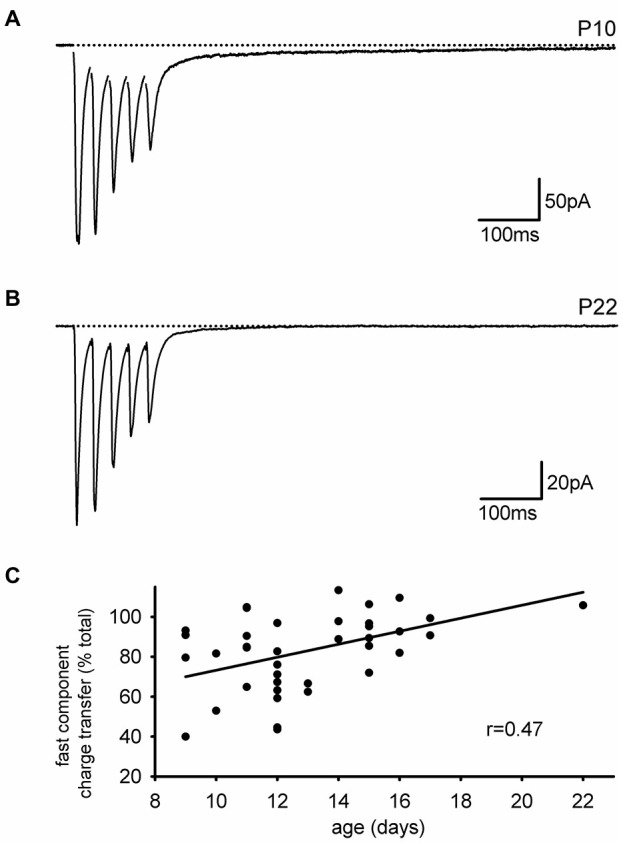
**The slow component of the EPSC is developmentally downregulated. (A)** Representative trace of an interneuronal EPSC from a neonatal mouse is shown. A large slow component is present. **(B)** Representative trace of an interneuronal EPSC from an older mouse with little slow component. **(C)** The contribution of the fast EPSC to the total charge transfer increases with animal age.

As we performed these experiments, we noted that the slow EPSC in our present set of experiments was smaller than expected based on our previous studies. One difference between the current results and previous ones is that the current set of experiments was done in mice while earlier studies were done in rats, both at about the same developmental stage (2–3 weeks). Reasoning that rats have a slower developmental entry into adulthood than mice, we considered that a developmental downregulation of the slow AMPAR EPSC might account for the smaller expression of the slow EPSC in mice. To test this idea directly, we divided the subjects by age (<P14 and ≥P14). Figures 5A,B display representative EPSCs in cells from neonatal and older mice. The neonatal group had a larger average SC as a fraction of the normalized integral (neonatal: *n* = 12, 14 ± 2.5%; juvenile: *n* = 12; 7.2 ± 1.4%, *p* < 0.05). A more quantitative analysis in which the size of the FC is expressed as a fraction of the total integral confirmed that the SC of the EPSC was developmentally downregulated with age (Figure 5C, *n* = 39, Pearson’s *r* = 0.472, *p* < 0.01).

### Developmental Downregulation of the Slow AMPAR EPSC is Independent of AMPAR Type

**Figure 6:**
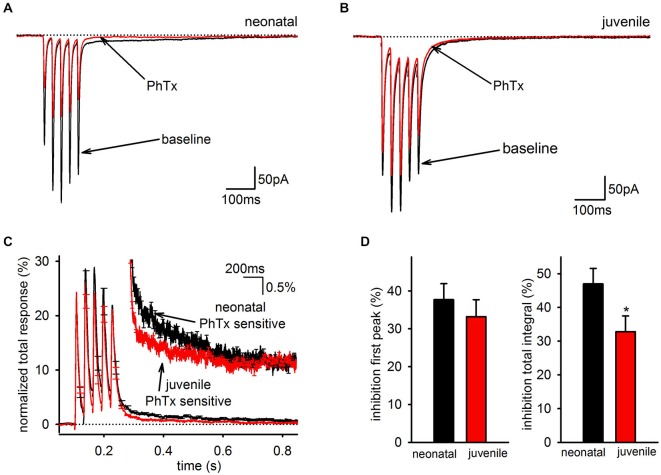
**The slow component of the GluA2-lacking AMPAR EPSC is developmentally downregulated. (A)** Representative average EPSCs before and after of bath application of PhTx are shown in an interneuron from a neonatal mouse. **(B)** Representative average EPSCs before and after bath application of PhTx in an interneuron from an older, juvenile mouse. **(C)** PhTx sensitive currents in neonatal and juvenile mice normalized to total response. Inset view of tail currents. Average kinetics of the slow component differs between neonatal and juvenile age groups. **(D)** Graph of average inhibition by PhTx in neonatal and juvenile animals. There was no difference in the inhibition of the first peak, but the total integral was inhibited to a greater degree in neonataler cells.

Because the SC of the EPSC is mediated predominantly by PhTx-sensitive AMPARs, the developmental downregulation of the slow EPSC could indicate any of three possible mechanisms: it could reflect a general decrease in synaptic GluA2-lacking AMPARs independent of whether they contribute to the fast or SC; it could reflect a general decrease in AMPARs mediating the SC independent of whether they contain GluA2; or it could reflect a selective of loss of only the GluA2-lacking AMPARs that mediate the slow EPSC. If there is a general decrease in GluA2 content of synaptic AMPARs, then PhTx should decrease in effectiveness over development for both the charge transfer of the EPSC, which includes both the fast and SCs of the EPSC, and the peak amplitude of the EPSC in response to the first stimulus pulse, which reflects only the FC. To differentiate between these possibilities, we compared PhTx sensitivity in neonatal (<P14) and juvenile (≥P14) animals (Figures 6A,B). When the kinetics of the PhTx-sensitive component from neonatal and juvenile animals were compared directly, it was clear that there was a selective loss of the slow EPSC relative to the fast EPSC in older animals (Figure 6C). There was no significant downregulation in the effectiveness of PhTx on the peak amplitude of the first EPSC (Figure 6D, left; neonatal: *n* = 12, 38 ± 4%; juvenile: *n* = 12, 33 ± 5%, *p* > 0.05), but PhTx had a much smaller effect on the total charge transfer in cells from older animals than it did in cells from neonatal animals (Figure 6D, right; neonatal: *n* = 12, 47 ± 5%; juvenile: *n* = 12, 33 ± 4%; *p* > 0.05). Thus, we conclude that the developmental downregulation of the slow AMPAR EPSC is not due to a general reduction in GluA2-lacking AMPARs but is specific to the AMPARs underlying the slow EPSC.

Does this selective downregulation of the slow EPSC affect only GluA2-lacking AMPARs, or does it also affect the GluA2-containing AMPARs that contribute to the slow EPSC? To answer this question, we examined the pharmacologically isolated GluA2-containing and—lacking currents for the neonatal and juvenile groups with bi-exponential fitting. Unsurprisingly, there was no age related difference between either the PhTx-resistant (neonatal: *n* = 12, 11 ± 1 ms; juvenile: *n* = 12, 13 ± 2 ms, *p* > 0.05) or the PhTx-sensitive (neonatal: *n* = 12, 10 ± 2 ms; juvenile: *n* = 12, 11 ± 1 ms, *p* < 0.05) fast time constants. Interestingly, there also was no difference in the PhTx-resistant (neonatal: *n* = 12, 319 ± 118 ms; juvenile: *n* = 12, 162 ± 56 ms, *p* < 0.05) or PhTx-sensitive (neonatal: *n* = 12, 550 ± 199 ms; juvenile: *n* = 12, 551 ± 110 ms, *p* < 0.05) slow time constants. However, both the PhTx-resistant (neonatal: *n* = 12, 19 ± 4%; juvenile: *n* = 12, 9 ± 2%, *p* < 0.05) and—sensitive (neonatal: *n* = 12, 17 ± 3%; juvenile: *n* = 12, 8 ± 2%, *p* < 0.05) SCs had a significantly reduced contribution to the biphasic EPSC in the juvenile group compared to the neonatal group. Therefore, the developmental regulation of the SC is independent of AMPAR composition and is due to a decrease in the relative size of the SC without changes in kinetics.

## Discussion

In the present study, we examined the role of GluA2 subunit-containing ionotropic glutamate receptors in generating the unusual biphasic EPSC observed in hippocampal interneurons at the SR/SLM border in area CA1. Two broad findings relate directly to the identity of the AMPAR subpopulations underlying the slow EPSC: first, we found that GluA2-lacking AMPARs preferentially contribute to the slow EPSC when uptake is intact, indicating the fast and slow EPSCs under normal conditions can be distinguished by their receptor subunit composition as well as kinetics. Second, the slow EPSC when uptake is intact is clearly distinct from the slow EPSC when EAAT1- mediated uptake is impaired, as the latter condition recruits an EPSC with vastly slower decay kinetics and no preferential contribution of GluA2-lacking AMPARs. These results indicate that the slow EPSC is disproportionately generated by a subpopulation of GluA2-lacking AMPARs distinct from the AMPARs underlying either the fast EPSC or from those underlying the very slow EPSC recruited by EAAT1 inhibition.

The observation that the slow EPSC is predominantly mediated by GluA2-lacking AMPARs provides a straightforward explanation for why this EPSC component is selectively observed in interneurons and not in neighboring pyramidal cells since the majority of their synaptically activated AMPARs contain the GluA2 subunit (Racca et al., 1996). However, the EPSCs mediated by GluA2-containing AMPARs and GluA2-lacking AMPARs both express a biphasic decay; the major difference between them was that this decay was much faster in GluA2-containing AMPARs than GluA2-lacking AMPARs, which is why the majority of the slow EPSC is mediated by GluA2-lacking AMPARs. Because the SC of each AMPAR subpopulation has not been observed in recordings of isolated AMPAR-mediated currents recordings from heterologous expression systems or from pyramidal cells, the inclusion or absence of GluA2 alone cannot explain the existence of the slow EPSC; rather, we suggest that some other factor is also required that slows the kinetics of AMPARs but does so more effectively for GluA2-lacking AMPARs. Since interneurons in general have a robust expression of GluA2-lacking AMPARs (Racca et al., 1996), the stronger expression of the slow EPSC in the SR/SLM than stratum oriens interneurons may reflect a cell-type specific difference in the expression of this factor (Nissen et al., 2010).

The identity of this factor remains unknown, but candidates would be members of the Transmembrane AMPA Regulatory Protein (TARP) family or cornichon proteins, both of which are known to influence AMPAR localization and kinetics (reviewed in Jackson and Nicoll, 2011). We note with interest that the cornichons and the TARP γ4 are both reported to profoundly prolong the decay kinetics of AMPAR-mediated currents. In fact, developmental changes in auxiliary proteins have been shown to alter the subunit composition and kinetics of AMPARs in hippocampal pyramidal cells (Blair et al., 2013). A second candidate would be post-translational modifications (i.e., phosphorylation, palmitoylation, and, ubiquitination). These modifications need not themselves be developmentally regulated, as their efficacy can depend on association of the AMPAR with a TARP (Kristensen et al., 2011). Finally, alterations in the splice variant present could underlie the observed shifts in AMPAR kinetics (Seifert et al., 2000). Future studies would be necessary to differentiate from these possibilities.

Although a slow EPSC can clearly be observed when glutamate uptake is intact, it is also clear that the activation of AMPARs on these cells is profoundly limited by the uptake of synaptically released glutamate. We previously reported that a concentration of TBOA sufficient to inhibit EAATs 1–3 recruits a slow AMPAR current that dominates the synaptic charge transfer; here this recruited AMPAR EPSC could be differentiated from the slow AMPAR EPSC in control conditions by far slower kinetics and the enhanced contribution of GluA2-containing AMPARs to the EPSC. In fact, the synaptic charge transfer of the EPSC in high doses of TBOA, which is dominated by the slow EPSC, was mediated predominantly by GluA2-containing AMPARs. Thus, the slow AMPAR EPSC in control conditions seems unlikely to reflect the same subpopulation of AMPARs as those recruited by high doses of TBOA. In contrast, lower doses of TBOA that preferentially inhibit EAATs 2–3 have a more modest effect, recruiting a slow EPSC similar in kinetics to the slow EPSC in control conditions. Selective inhibition of EAAT2 with WAY213613 had no effect, suggesting that EAAT2 does not contribute substantively to glutamate uptake at these synapses; this conclusion is somewhat unexpected given the known contribution of EAATs 1–2 at the neighboring Schaffer collateral synapses (Bergles and Jahr, 1998), but may indicate that the localization of EAAT2 is directed more effectively to glutamatergic synapses onto pyramidal cells than those onto interneurons.

Because the differential effects of high and low doses of TBOA primarily reflect greater inhibition of EAAT1, and EAAT1 is localized outside of the synaptic cleft on glial cells, we suggest that the AMPAR EPSC recruited by high doses of TBOA represents the activation of extrasynaptic AMPARs that predominantly express GluA2-containing AMPARs normally precluded from activation by glial EAAT1. We note that this is the opposite to pyramidal cells, where extrasynaptic receptors predominantly lack GluA2 (Zamanillo et al., 1999); presumably this is another facet of the differences between these cell types in trafficking and expression of GluA2-containing and GluA2-lacking AMPARs. As the low doses of TBOA in these experiments should primarily reflect inhibition of neuronally-localized EAAT3 (Mennerick et al., 1998; Holmseth et al., 2012), one possible interpretation is that EAAT3 inhibition affects the spread of glutamate within the synaptic cleft and leads to more effective recruitment of synaptic AMPARs to the slow EPSC. Unfortunately, the AMPARs recruited by low TBOA are difficult to define because their kinetics overlap with the slow EPSC in the absence of TBOA, and because the pharmacological distinction between 10 μM and 100 μM TBOA is a preferential but not exclusive inhibition of neuronal EAAT3 over glial EAAT1. We presume that this latter issue explains the few cells in which the very slow EPSC, reliably observed at high doses of TBOA, could be detected at the low dose of TBOA. Thus, we caution that a more selective means of affecting EAAT3 and not EAAT1 will be required to more definitively assess the role of EAAT3 in limiting the size of the slow EPSC.

**Figure 7:**
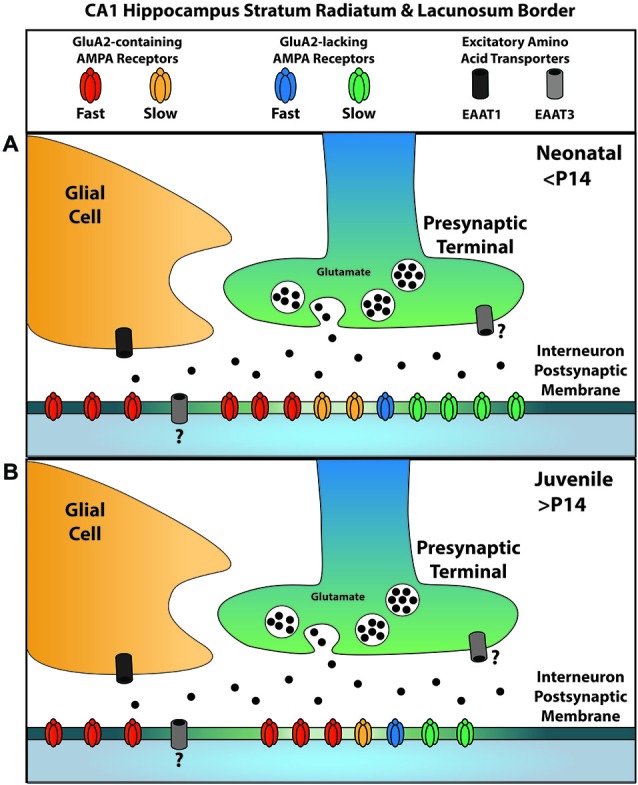
**A summary of AMPAR and EAAT localizations for glutamatergic synapses onto SR/SLM interneurons**. Synaptic AMPARs can be subdivided into two kinetically distinct forms, one with slow kinetics and the other with fast kinetics; similarly, they can be subdivided according to the presence or absence of GluA2. **(A)** Among the receptors with fast kinetics, GluA2-containing AMPARs predominate; among those with slow kinetics, GluA2-lacking AMPARs predominate. EAAT1, a glial glutamate transporter, is primarily responsible for the uptake that suppresses glutamate spill-over to activate extrasynaptic AMPARs; these extrasynaptic AMPARs predominantly contain GluA2. EAAT3 is also present, but plays a lesser role in limiting spillover. The question mark denotes uncertainty in the localization of this transporter at this synapse. **(B)** Over the course of development, AMPARs with slow kinetics are removed from the synapse independent of their GluA2 content. The relative proportion of receptors represents the contribution of each subtype to the overall charge transfer and does not presume to represent the actual population number due to the influence of subtype specific parameters such as conductance.

Our past and continuing experiments allow us to more completely define the localization of glutamatergic signaling elements at the excitatory synapses on GABAergic interneurons along the SR/SLM border of the hippocampus with respect to their location relative to the active zone (Figure 7). GluK1-containing KARs are localized to the synaptic cleft with no appreciable presence outside it (Wondolowski and Frerking, 2009). Within the synapse, GluA2-containing AMPARs preferentially contribute to the fast AMPAR EPSC, while GluA2-lacking AMPARs preferentially contribute to the slow AMPAR EPSC; however, both receptors contribute to both kinetic components of the EPSC at least to some degree. Extrasynaptic receptors predominantly are GluA2-containing AMPARs and are not normally activated by synaptic glutamate because EAAT1 in nearby glia effectively prevents spillover. EAAT3 may also limit the activation of AMPARs within the synaptic cleft, although it remains unclear whether this transporter is located presynaptically or postsynaptically. We also note that Figure 7 reflects the localization of receptors and transporters relative to the synaptic cleft, and for simplicity does not reflect the apparent segregation of AMPARs with fast and slow kinetics to different synapses (Wondolowski and Frerking, 2009).

Finally, we report here that the expression of the slow AMPAR EPSC is developmentally downregulated, with limited expression remaining after 3 weeks of postnatal development in mice. A developmental shift in the opposite direction has been seen in pyramidal cells, where AMPA responses were prolonged after 3 weeks of age in rats by an alteration in AMPAR subunit composition and an increase in TARP expression (Blair et al., 2013). For both the effect observed here, GluA2-containing and—lacking AMPAR EPSCs, the kinetics of the SCs remained constant while the overall contribution of the SC to the EPSC decreased. This suggests that AMPARs with slow kinetics are selectively removed from the synapse over the course of development, independent of GluA2 content. Whether this kinetic effect reflects a change in other subunits underlying the AMPAR EPSC or a change in the complement of accessory proteins remains unclear. Regardless of the mechanisms involved, the developmental downregulation of the slow AMPAR EPSC suggests that the proposed role of interneurons in mediating temporally precise forms of information processing (McBain et al., 1999) is likely to be limited at birth and become more prominent over postnatal development, at least in this subpopulation of cells. Such a transient window of broadly-tuned temporal integration followed by more precise coincidence detection could assist in the initial formation and subsequent refinement of hippocampal circuitry during early development (Calixto et al., 2008). The calcium permeable nature of GluA2-lacking AMPARs also raises the possibility that this subpopulation of receptors could play a role in synaptic plasticity (Wiltgen et al., 2010).

In summary, our results indicate that the kinetic components of the AMPAR EPSC on hippocampal SR/SLM interneurons are mediated by distinct subpopulations of AMPARs that differ in their GluA2 expression, with GluA2-lacking AMPARs preferentially contributing to the slow AMPAR EPSC under normal conditions and GluA2-containing AMPARs preferentially contributing to the fast AMPAR EPSC. The slow EPSC can be differentiated from the spillover-mediated EPSC that is suppressed by EAAT1-dependent glutamate uptake, and is also developmentally downregulated. These results collectively suggest that the subunit composition of AMPARs is a major factor influencing the kinetics and charge transfer of the slow EPSC, thereby regulating the contribution of AMPAR-mediated transmission to temporal precision in interneuronal signaling.

## Conflict of Interest Statement

The authors declare that the research was conducted in the absence of any commercial or financial relationships that could be construed as a potential conflict of interest.
